# Targeted nanoparticle-mediated Co-delivery of IFITM3 KO and ponatinib reverses TKI resistance in chronic myeloid leukemia

**DOI:** 10.3389/fphar.2026.1852978

**Published:** 2026-07-28

**Authors:** Zhenyang Feng, Yaofang Ma, Jiahui Hu, Yan Wang, Kexin Wang, Ying Yang, Qiyuan Wu, Jiatian Lou, Ruoyuan Shi, Kejia Xu, Wanlin Yang, Li Li, Linglan Tu, Liyan Cheng

**Affiliations:** 1 School of Laboratory Medicine and Bioengineering, Hangzhou Medical College, Hangzhou, Zhejiang, China; 2 Clinical Medical College, Hangzhou Medical College, Hangzhou, Zhejiang, China; 3 Kangda College, Nanjing Medical University, Lianyungang, Jiangsu, China; 4 Zhejiang Key Laboratory of Tumor Molecular Diagnosis and Individualized Medicine, Hangzhou Medical College, Hangzhou, Zhejiang, China; 5 Huzhou Municipal Hospital of Traditional Chinese Medicine, Huzhou, Zhejiang, China

**Keywords:** chronic myeloid leukemia, drug resistance, IFITM3, nanoparticle delivery system, ponatinib

## Abstract

Resistance to tyrosine kinase inhibitors (TKIs) is a core limitation in the clinical treatment of chronic myeloid leukemia (CML). Although ponatinib can cover the T315I mutation, its clinical application is limited by severe adverse reactions at high doses. On the basis of the USP28-BCR-ABL-IFITM3 resistance signaling axis first identified in our previous study, a ginger-derived lipid carrier-mediated targeted nanodelivery system (IP@GLPs@εF) was constructed, which codelivers CRISPR/Cas9-mediated IFITM3 knockout (IFITM3 KO) plasmid and ponatinib, establishing a new synergistic intervention mode of gene editing and targeted chemotherapy. When modified with ε-polylysine and fucoidan via layer-by-layer self-assembly technology, the carrier has an average particle size of 226.1 nm, a drug encapsulation efficiency of 84.2%, and excellent biocompatibility. *In vitro* experiments confirmed that the optimal ratio (2.5 μg IFITM3-sg3 + 5 μM ponatinib) significantly reversed the drug resistance of K562R cells, promoted apoptosis and inhibited proliferation. *In vivo* experiments using ectopic and orthotopic xenograft models verified that this system can efficiently target tumor tissues and significantly suppress the progression and metastasis of drug-resistant tumors, with no obvious toxic side effects on major organs. Mechanistically, this study revealed that IFITM3 mediates CML resistance by interacting with HSPA9 to activate the MET/AKT/BCL2 pathway and that IFITM3 KO can block this pathway and exert a synergistic antiresistance effect with ponatinib. This research provides a novel IFITM3-targeted synergistic therapeutic strategy and technical support for the clinical treatment of CML resistance.

## Graphical Abstract

**Figure:**
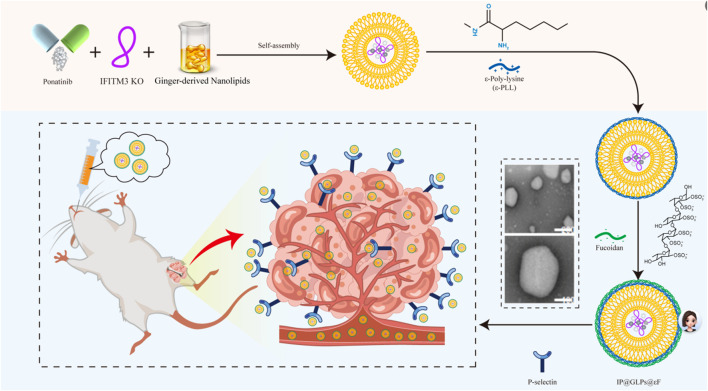

## 1 Introduction

CML is a malignant clonal proliferative disorder originating from haematopoietic stem cells. The widespread clinical application of TKIs has significantly improved the survival outcomes of CML patients (Jabbour and Kantarjian, 2025), yet drug resistance remains a central bottleneck in current clinical management (Jung et al., 2025; Jabbour and Kantarjian, 2024). Acquired resistance, the most prevalent subtype of CML, is characterized by gradual attenuation of TKI efficacy, accompanied by progressive deterioration of haematological, cytogenetic, and molecular parameters. Its underlying mechanisms are complex and multifactorial, encompassing diverse molecular events such as BCR-ABL1 kinase domain mutations, gene copy number amplification, hyperactivation of drug efflux pumps, and aberrant activation of intracellular signaling cascades (Sun C. et al., 2025; Soverini and Castagnetti, 2025; Tang et al., 2025; Alekrish et al., 2025). Clinical evidence indicates that the 5-year cumulative incidence of resistance reaches 20%–30% in CML patients treated with imatinib, a first-generation TKI (Jabbour and Kantarjian, 2025). To address this intractable challenge, third-generation TKIs, typified by ponatinib, have been developed for the targeted treatment of Philadelphia chromosome-positive (Ph^+^) CML patients harbouring the T315I mutation. However, high-dose ponatinib is associated with substantial adverse effects, and its clinical use is limited by stringent indications (Kantarjian et al., 2025; Schenk et al., 2026; Devos et al., 2025). Thus, strategies to reduce ponatinib dosage, achieve highly precise tumor targeting, and thereby optimize therapeutic efficacy while ensuring safety have emerged as pivotal research focuses and key hurdles in this field (Devos et al., 2025; Sadaga et al., 2025). In this context, this study focused on BCR-ABL1-dependent CML drug resistance mechanisms and intervention strategies. Our previous work confirmed that IFITM3 is markedly upregulated in BCR-ABL-dependent imatinib-resistant CML cell lines and that imatinib resistance may be driven by activation of the USP28-BCR-ABL-IFITM3 signaling axis (Li et al., 2024). Moreover, the role of IFITM3 in the drug resistance of leukemia and solid tumors has attracted increasing attention, with relevant studies being extensively conducted and further advanced (Cui Y. et al., 2025; Ibusuki et al., 2025; Chu et al., 2022; Xiong et al., 2024). Building on these preliminary findings, we constructed a self-developed dual-loading nanoparticle delivery system to achieve synergistic targeted codelivery of CRISPR/Cas9-mediated IFITM3 knockout (IFITM3KO) and the third-generation TKI ponatinib. This delivery system enables IFITM3 genome editing to block resistance signaling at the source and reduce compensatory pathway activation, with improved target specificity and safety. Compared with conventional synthetic lipid nanoparticles (LNPs), ginger-derived GLPs show excellent biocompatibility and low immunogenicity, allow low-cost large-scale preparation, and support efficient co-delivery of hydrophobic ponatinib and negatively charged CRISPR plasmids via facile layer-by-layer targeting modification. Through systematic pharmacodynamic evaluation and molecular mechanism elucidation, we explored the synergistic therapeutic advantages of combined gene editing and targeted chemotherapy, aiming to provide an innovative theoretical basis and technical approach for the clinical management of drug-resistant CML.

## 2 Materials and methods

### 2.1 Cell culture and transfection

The K562 cell line was preserved in our laboratory, and the imatinib-resistant K562 R subline was established from parental imatinib-sensitive K562 cells via continuous drug induction. Briefly, K562 cells were cultured in RPMI-1640 medium with gradually elevated imatinib concentrations ranging from 0.5 µM to 8 µM for 12 months. The imatinib dose was increased by 0.5 μM at each interval and maintained for 15–30 days according to cell growth status, finally constructing the K562R cell line with stable resistance to 8 µM imatinib. Identification of drug resistance was accomplished.

Both K562 and K562 R cells were cultured in RPMI-1640 medium (Gibco, Grand Island, NY, United States) supplemented with 10% fetal bovine serum (FBS; HyClone, Logan, UT, United States), and incubated at 37 °C in a humidified atmosphere containing 5% CO_2_.

Three distinct sgRNA sequences targeting the human IFITM3 gene were designed and namedsg1 (GTGAAGGTGCGTATGGCCCC),sg2 (GCG​CTC​TGA​GGA​GCT​CCC​TG), and sg3 (GTG​CTG​ATC​TTC​CAG​GCC​TA). The IFITM3 overexpression plasmid was purchased from WZ Bioscience (Shandong, China). Cell transfection was conducted using Lip 2000 (Cat. No. BL623B; Biosharp, Anhui, China) and Opti-MEM (Gibco, United States). Cells were collected for subsequent experiments at least 48 h after transfection.

### 2.2 Isolation and purification of ginger-derived exosome-like nanoparticles

Fresh ginger rhizomes were thoroughly cleaned, peeled and homogenized for juice extraction. The obtained ginger juice mixture was subjected to filtration, followed by sequential centrifugation steps: first at 1,000–200×g for 10–30 min; the resultant supernatant was further centrifuged at 4,000–5,000×g for 30–40 min; the collected supernatant was spun at 8,000–10000×g for 40–60 min. Afterwards, the supernatant was ultracentrifuged at 120000–150000×g for 120–150 min, and the sediment was resuspended in sterile PBS via sonication to prepare ginger-derived liposomal suspension. Subsequently, density-gradient purification was performed using a stepwise sucrose gradient (8%, 30%, 45%, and 60%, w/v) by ultracentrifugation at 120000–150000 × g for 120–150 min. Finally, the purified exosome-like nanoparticles were preserved at −80 °C for subsequent experiments.

### 2.3 Isolation and purification of ginger phospholipids

Ginger-derived phospholipids were extracted and purified via a modified Bligh-Dyer method. Briefly, methanol/chloroform mixture was added to ginger-derived exosome-like nanoparticles at a volumetric ratio of 2:1, followed by vigorous vortexing. An equal volume of chloroform and deionized water was supplemented sequentially; after another vortex, the mixture was centrifuged at 1000–1200×g to trigger phase separation. The organic phase was harvested to obtain target ginger-derived phospholipids.

### 2.4 Surface functionalization of ginger-engineered lipid carriers

The prefabricated ginger-engineered lipid carriers were resuspended and diluted to a concentration of 1 mg/mL using pH-adjusted phosphate-buffered saline (PBS, pH 6.0–8.0). Meanwhile, fresh 1 mg/mL PBS solutions of ε-polylysine and fucoidan were prepared for immediate use. Based on the preset phospholipid/ε-PL/fucoidan mass ratios ranging from 10:1:0.5 to 10:3:2, the ε-polylysine solution was introduced into the carrier suspension, followed by continuous stirring at 200–600 r/min for 15–60 min at room temperature. Subsequently, an equal corresponding volume of fucoidan solution was added, and the stirring reaction was maintained for an additional 15–60 min. Utilizing electrostatic interaction-driven layer-by-layer (LbL) self-assembly, the targeted functional lipid carriers were successfully fabricated.

The resulting reaction mixture was ultracentrifuged at 100,000×g for 60 min at 4 °C. The collected precipitate was resuspended in PBS and washed twice to eliminate unbound residual components. Finally, orthogonal experiments combined with analysis of variance (ANOVA) were performed to screen and determine the optimal functional modification conditions, which satisfied the criteria of stable zeta potential, fucoidan binding efficiency exceeding 80%, and polydispersity index (PDI) lower than 0.3.

### 2.5 Characterization and optimization of the physicochemical properties of ginger-engineered lipid carriers

The morphological characteristics of synthesized nanoparticles were observed by transmission electron microscopy. Particle size, polydispersity index (PDI), and zeta potential were measured using a Malvern nanoparticle size analyzer. High-performance liquid chromatography (HPLC) was applied to quantify the encapsulation efficiency (EE) and drug loading capacity (LC). Nanoparticle stability was evaluated via 0–30 days of storage at 4 °C and 37 °C, with regular detection of changes in particle size and PDI. The optimal N/P ratio (1:1 to 10:1) between transfection reagents and plasmids was screened based on cellular transfection efficiency. Dialysis assays in buffers with pH values of 5.0, 6.5, and 7.4 were performed to investigate the *in vitro* sustained drug release behavior.

Orthogonal tests combined with analysis of variance (ANOVA) were utilized to determine the optimal nanoparticle preparation conditions. The core screening criteria included a stable zeta potential, fucoidan binding efficiency above 80%, PDI below 0.3, encapsulation efficiency and drug loading capacity exceeding 80% and 8%, respectively, as well as satisfactory storage stability and sustained drug release performance.

### 2.6 Preparation of ginger-engineered lipid carriers coloaded with the IFITM3-KO plasmid and ponatinib

Ginger-engineered lipid carriers loaded with drugs were prepared via the thin-film ultrasonic method. The IFITM3-KO plasmid was mixed with an appropriate amount of TurboFect™ transfection reagent to form a plasmid/transfection reagent complex. Chloroform in the phospholipid components of ginger was removed by rotary evaporation under nitrogen protection, after which the plasmid/transfection reagent complex and ponatinib were added at a specific ratio. Ginger-engineered lipid carriers coloaded with the IFITM3-KO plasmid and ponatinib were prepared under ultrasonic treatment, and finally, the lipid carriers were purified and collected by ultracentrifugation.

### 2.7 Evaluation of the synergistic enhancement effect of the IFITM3-KO plasmid and ponatinib

CML cells were cocultured with functionalized coloaded formulations at various concentrations, and a CCK-8 assay was used to evaluate the effect of the aforementioned formulations on CML cell proliferation at different time points under distinct treatment conditions to verify the synergistic enhancing effect of the IFITM3-KO plasmid and the targeted drug.

### 2.8 Evaluation of tumor cell apoptosis

The functionalized coloaded drug carriers were coincubated with K562 R cells. The apoptosis of cells under different treatment conditions was detected via flow cytometry using an Annexin V-FITC/PI apoptosis detection kit to verify the proapoptotic effect of lipid carriers coloaded with IFITM3-KO plasmids and ponatinib on drug-resistant CML cells.

### 2.9 Establishment of a CML xenograft tumor model and treatment regimen

Log-phase K562R cells were harvested, and 5 × 10^6^ cells were mixed with Matrigel at a 1:1 volume ratio. The tumor cell/Matrigel suspension was subcutaneously injected into the axilla of the nude mice. Four weeks post-injection, the tumor-bearing mice were randomly assigned to four groups: control, ponatinib monotherapy, IFITM3 KO/ponatinib@LPs@εF (IP@LPs@εF), and IFITM3 KO/ponatinib@GLPs@εF (IP@GLPs@εF). All formulations were orally gavaged at 10 mg/kg for 5 consecutive days/week for 2 weeks.

### 2.10 Establishment of a CML orthotopic mouse model and administration regimen

The log-phase K562 R cell line was harvested and injected via the tail vein at 1 × 10^6^ cells/mouse. Two weeks post-injection, the model mice were randomly assigned to four groups: control, ponatinib monotherapy, IFITM3 KO/ponatinib@LPs@εF (IP@LPs@εF), and IFITM3 KO/ponatinib@GLPs@εF (IP@GLPs@εF). All formulations were orally gavaged at 10 mg/kg for 5 consecutive days/week for 2 weeks. The nanoparticles were administered to mice in a colloidal dispersion state. Pure water was used as the dispersion medium.

### 2.11 Tumor spheroid uptake and imaging assay

Functionalized and nonfunctionalized dual-drug carriers were labeled with DIL fluorescent dye by incubation at 37 °C in the dark for 30–60 min. Free dye was removed via centrifugation, followed by washing and resuspension. K562R cells were adjusted to 2 × 10^3^ cells/well, mixed thoroughly with 1:3 diluted Matrigel, and seeded into ultralow-attachment 96-well plates. The plates were first incubated at 37 °C for 15–30 min to induce rapid Matrigel gelation (providing an adhesive scaffold for cell aggregation and regular 3D tumor spheroid formation). Labeled drugs at the same final concentration were then added to both spheroid groups, which were subsequently incubated at 37 °C with 5% CO_2_ in the dark for 4–24 h, after which they were washed and subjected to fluorescence-brightfield colocalization imaging under a confocal laser scanning microscope (CLSM).

### 2.12 Biosafety evaluation

Each group of drugs was administered continuously at the normal dosage for 1 month. The major organs and whole blood of the mice in each experimental group were collected. The major organs were subjected to H&E staining and weight measurement, while the whole blood was analysed for biochemical indicators. The biosafety of the functionalized lipid carrier drugs was evaluated on the basis of the above results.

### 2.13 Data collection

We analysed the expression patterns of the IFITM3 and HSPA9 genes in leukemia using data from The Cancer Genome Atlas (TCGA) database (https://portal.gdc.cancer.gov/). Data analysis results from the University of Alabama at Birmingham Cancer Data Analysis Portal (UALCAN) (https://ualcan.path.uab.edu/index.html) revealed that HSPA9 is significantly upregulated in leukemia and that its expression is positively correlated with the number of Philadelphia chromosome-positive cells (Pearson correlation coefficient = 0.51).

### 2.14 Statistical analysis

GraphPad Prism 9 (San Diego, CA, United States) was used for statistical analysis in this study. The results are expressed as the means ± standard deviations (SDs). A t-test was employed to conduct comparative analysis on two sets of data, and analysis of variance was used for comparisons among multiple sets of data. All the above *in vitro* experiments were repeated three times. A p value less than or equal to 0.05 was considered to indicate statistical significance. The significance level is indicated by asterisks: * indicates p ≤ 0.05, ** indicates p < 0.01, *** indicates p < 0.001, and **** indicates p < 0.0001. P > 0.05 is indicated as N.S. (not significant).

## 3 Results

### 3.1 Characterization of drugs self-assembled from ginger-derived lipid vehicles

**FIGURE 1:**
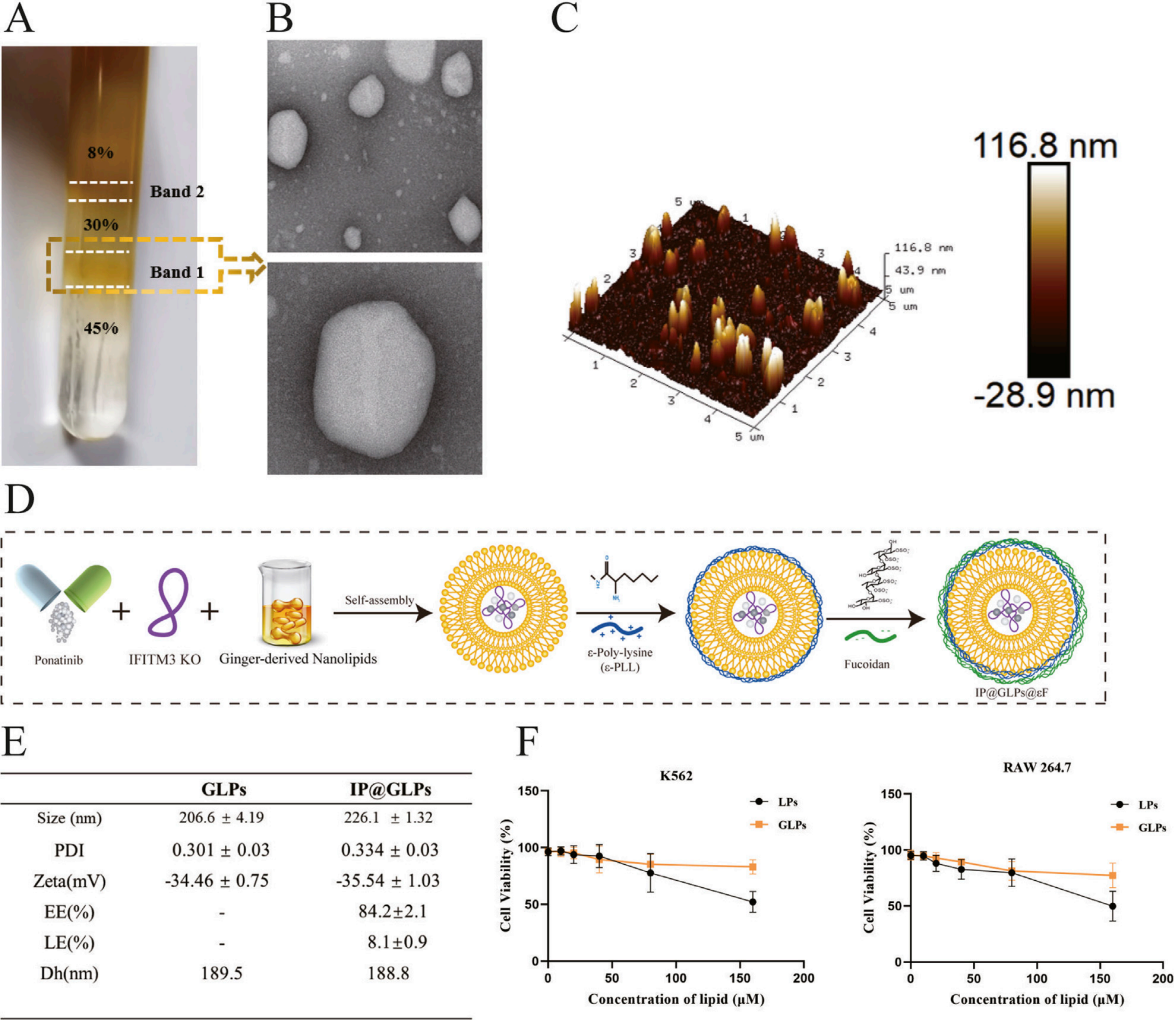
Characterization of ginger-derived lipid vehicle self-assembly with drugs. **(A)** The band 1 fraction isolated from the 30%/45% interface (yellow rectangle) was designated ginger-derived nanolipids (GLPs). **(B,C)** Morphological characterization of the GLPs via TEM **(B)** and AFM **(C)**. **(D)** Schematic of the self-assembly and fabrication of the IP@GLPs@F drug-loading system. **(E)** Physicochemical properties of the GLPs and IP@GLPs. **(F)** Biocompatibility of the GLPs. All the data are presented as the means ± SDs of three independent experiments.

To validate the self-assembly capability and delivery potential of ginger-derived exosome-like lipids, nanoparticles were fabricated via conventional lipid film hydration. Transmission electron microscopy (TEM, Figures 1A,B) and atomic force microscopy (AFM, Figure 1C) verified the uniform nanoscale spherical morphology of ginger lipid particles (GLPs). Dynamic light scattering (DLS) measurements (Figure 1E) showed that GLPs exhibited a mean hydrodynamic diameter of 189.5 nm, a polydispersity index (PDI) of 0.301, and a zeta potential of −34.46 mV.

Subsequently, GLPs were co-loaded with ponatinib and IFITM3-KO plasmids, followed by sequential modification with ε-PLL and fucoidan to construct dual-functional IP@GLPs@F nanocarriers (Figure 1D). DLS characterization demonstrated that IP@GLPs displayed negligible variations in particle size (226.1 nm), PDI (0.334) and zeta potential (−35.54 mV) compared with blank GLPs, with a favorable ponatinib encapsulation efficiency of 84.2% (Figure 1E). A CCK-8 assay was performed to evaluate the biocompatibility of GLPs in RAW 264.7 and K562 cells, with commercial liposomes (LPs) set as the control group. After 24 h of incubation, GLPs exerted minimal cytotoxicity to both cell lines. In contrast, commercial LPs impaired cell viability in a lipid concentration-dependent manner (Figure 1F).

Collectively, ginger-derived exosome-like lipids can self-assemble into uniform nanoparticles with excellent biocompatibility, serving as a promising versatile platform for small-molecule drug and nucleic acid co-delivery.

### 3.2 Optimal dosage ratio of the IFITM3 KO plasmid-ponatinib combination

**FIGURE 2:**
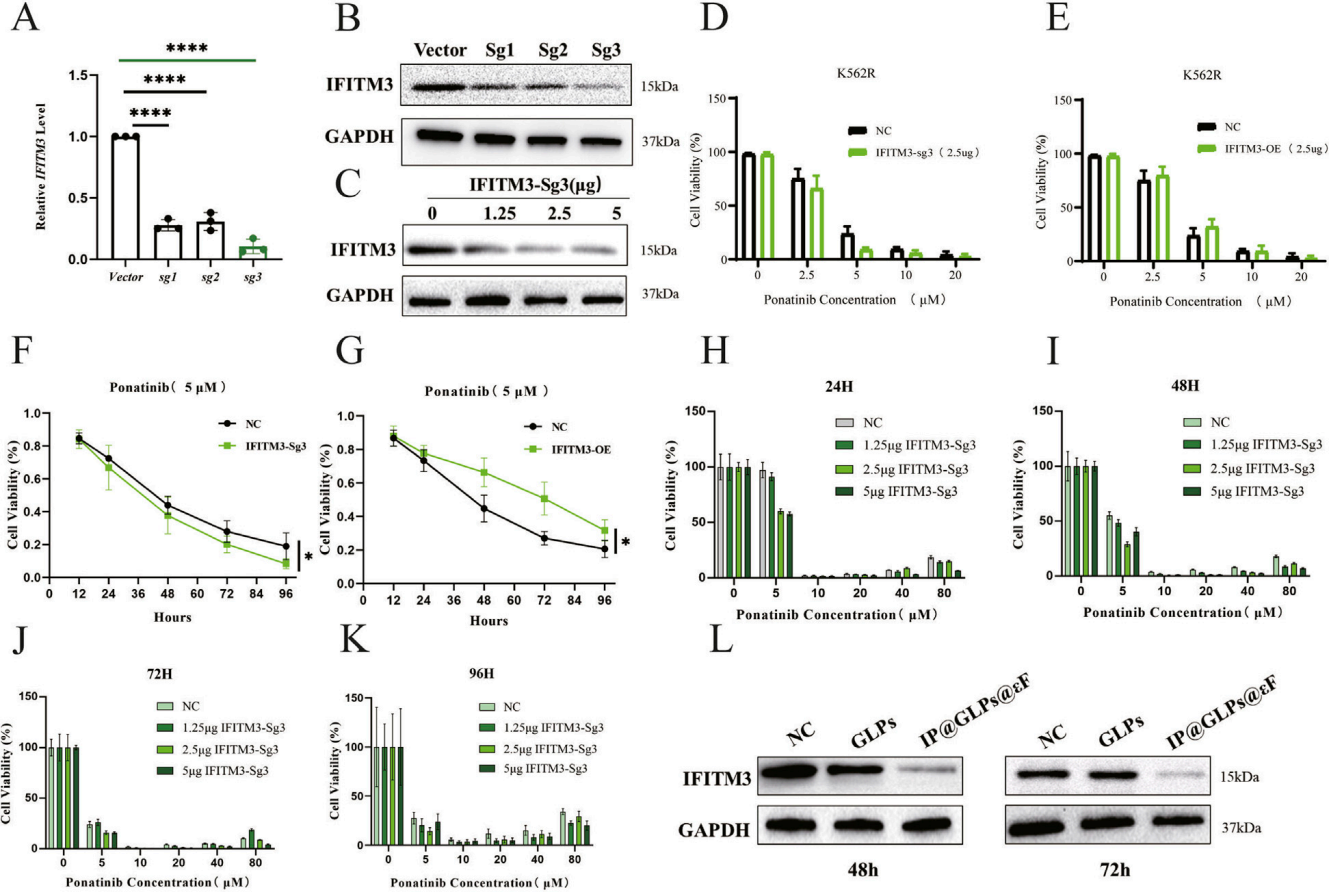
Optimal dosage ratio of the IFITM3-KO plasmid-ponatinib. **(A)** IFITM3 mRNA expression in K562 R cells transfected with control vector or IFITM3-sg1/2/3 was measured by qRT‒PCR (normalized to that of GAPDH). **(B,C)** IFITM3 protein knockdown efficiency in K562 R cells transfected with IFITM3-sg1/2/3 **(B)** or gradient doses of IFITM3-sg3 **(C)** analysed via Western blot. **(D,E)** Viability of K562 R cells treated with gradient concentrations of ponatinib combined with 2.5 μg IFITM3-sg3 **(D)** or IFITM3-OE **(E)** as measured via CCK-8 assay. **(F,G)** Time course viability of K562 R cells treated with 5 μM ponatinib combined with IFITM3-OE **(F)** or IFITM3-sg **(G)**, as detected via CCK-8 assay. **(H–K)** Viability of K562R cells treated with ponatinib (0–80 μM) combined with 1.25 μg, 2 μg or 5 μg of IFITM3-sg3 at 24 h **(H)**, 48 h **(I)**, 72 h **(J)** and 96 h **(K)** determined via CCK-8 assay. **(L)** IFITM3 protein expression in K562 R cells from different treatment groups analysed via Western blotting. All the data are presented as the means ± SDs of three independent experiments (*p < 0.05, **p < 0.01, ***p < 0.001, ****p < 0.0001, N.S. P > 0.05).

Three sgRNAs targeting human IFITM3 were designed for CRISPR-Cas9-mediated gene editing. PCR screening verified that IFITM3-sg3 yielded markedly superior knockdown efficiency compared with sg1 and sg2 (Figure 2A). Western blot analysis further confirmed that all three sgRNAs successfully downregulated endogenous IFITM3 protein expression in K562 cells (Figure 2B). A plasmid dose-gradient assay (1.25 μg, 2.5 μg, and 5 μg) demonstrated that 2.5 μg IFITM3-sg3 achieved robust and optimal IFITM3 silencing (Figure 2C).

In drug-resistant K562R cells, IFITM3 knockdown synergistically enhanced ponatinib cytotoxicity in a dose-dependent manner. With increased ponatinib administration, cells treated with 2.5 μg IFITM3-sg3 exhibited significantly lower viability than control cells (Figure 2D). This synergistic anti-tumor effect was also time-dependent. Under 5 μM ponatinib stimulation, the IFITM3-sg3 group showed continuous cell viability decline across all time points relative to controls (Figure 2F). In contrast, IFITM3 overexpression abrogated this therapeutic effect and notably restored cell viability under identical ponatinib treatment (Figures 2E,G).

Multitime-point CCK-8 assays were performed to evaluate sustained therapeutic efficacy. At 24 h post-treatment (Figure 2H), low-dose ponatinib caused negligible cytotoxicity in all groups, with no obvious intergroup differences, while high-dose ponatinib mildly suppressed cell viability. At 48 h (Figure 2I) and 72 h (Figure 2J), high-concentration ponatinib exerted enhanced cytotoxicity, and the combination of 2.5 μg IFITM3-sg3 and 5 μM ponatinib produced the most potent cell growth inhibition. After 96 h of treatment (Figure 2K), high-dose ponatinib induced profound cell death across all experimental groups.

Subsequently, optimal-dose IFITM3-sg3 and ponatinib were co-encapsulated into functionalized ginger-derived lipid carriers. Western blot results validated that IP@GLPs@εF significantly downregulated IFITM3 protein expression in K562R cells compared with blank GLPs (Figure 2L). Collectively, the combination of 2.5 μg IFITM3-sg3 and 5 μM ponatinib was identified as the optimal therapeutic regimen, which could be efficiently delivered via engineered ginger lipid nanocarriers, laying a solid foundation for subsequent mechanistic and functional experiments.

### 3.3 Evaluation of the cellular uptake and tumor-targeting ability of the dual-drug delivery system

**FIGURE 3:**
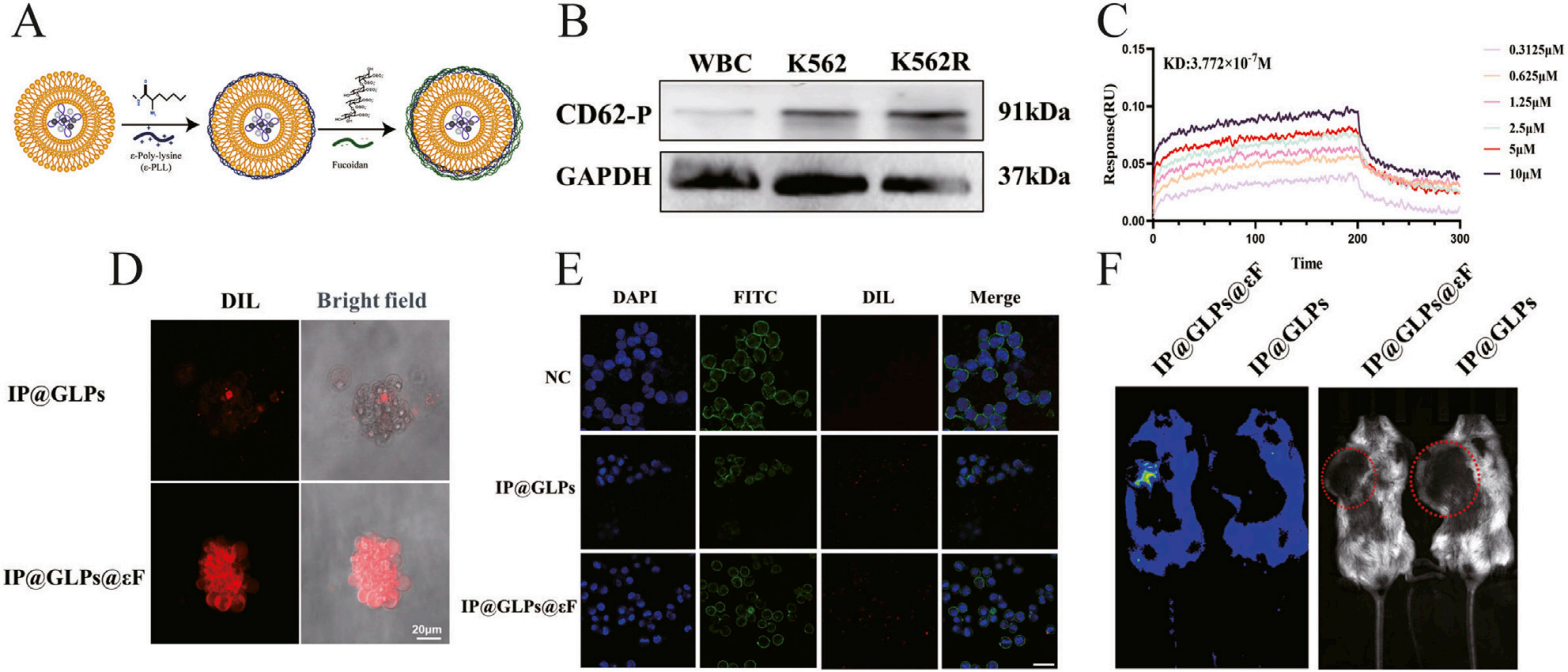
Cellular uptake and tumor targeting of the dual-drug delivery system. **(A)** Schematic illustration of functionalized lipid carriers formed by Zingiber lipidosomes encapsulating fucoidan via electrostatic adsorption. **(B)** Western blot analysis verifying P-selectin expression levels in K562 and K562R cells. **(C)** Curves of the binding of fucoidan to P-selectin, validated by biolayer interferometry (BLI). **(D)** In vitro tumor spheroid assay for evaluating the cellular uptake capacity of functionalized lipid carriers. **(E)** Fluorescence immunostaining assay verifying the cellular uptake of the dual-loaded drugs. **(F)** In vivo imaging for detecting the tumor-targeted distribution of lipid carriers in tumor-bearing mice.

**FIGURE 4:**
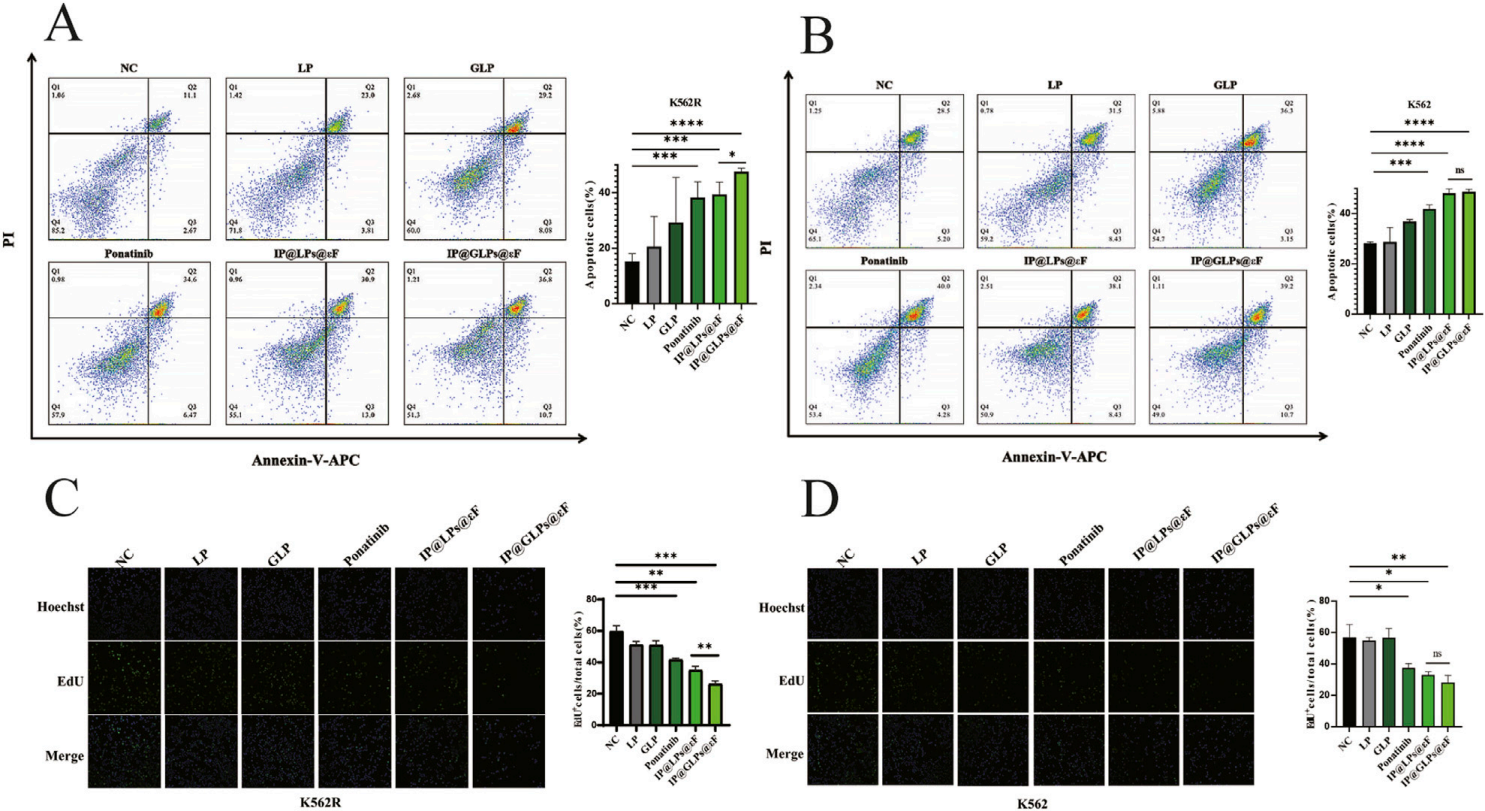
Resistant leukemia cell viability affected by the dual-drug system. **(A,B)** Results of the Annexin V-PI flow cytometry apoptosis assay (left: original flow cytometry plot; right: quantitative analysis of apoptotic cells). **(C,D)** Results of the EdU proliferation assay (left: fluorescence images of Hoechst/EdU staining; right: quantitative analysis of EdU-positive cells). All the data represent the means ± SDs from three independent experiments. *p < 0.05, **p < 0.01, ***p < 0.001, ****p < 0.0001, N.S. P > 0.05.

Given the electronegativity of ginger-engineered lipid carriers, positively charged ε-polylysine and negatively charged fucoidan were sequentially modified on nanolipid carriers via layer-by-layer assembly, yielding P-selectin-targeted ginger-engineered lipid carriers (functionalized liposomes) (Figure 3A). Western blot analysis revealed high CD62-P expression in leukemia cells, which was further upregulated in the drug-resistant K562 R line (Figure 3B). Biolayer interferometry (BLI) confirmed targeted binding between P-selectin and fucoidan. Fucoidan (0.3125–10 μM) bound to P-selectin, with the binding response increasing with increasing concentration. The dissociation constant (K) was 3.772 × 10^−7^ M, indicating strong targeting affinity (Figure 3C). DIL fluorescence labeling combined with fluorescence-bright field colocalization was used to assess the delivery of IP@GLPs and εF-modified IP@GLPs@εF to K562 R tumor spheroids. Unmodified IP@GLPs showed sparse, weak fluorescence with partial coverage and poor penetration, whereas IP@GLPs@εF exhibited intense, concentrated fluorescence covering the entire spheroid (Figure 3D). Coincubation of the functionalized liposomes with the target cells resulted in significantly greater cellular uptake at 48 h than that of the unfunctionalized liposomes (Figure 3E). In a mouse model of ectopic large tumors, ginger liposomes loaded with drugs exhibited stronger fluorescence in tumor tissues at 8 h postadministration, confirming enhanced tumor accumulation and superior penetration (Figure 4F).

### 3.4 Effect of the dual-drug delivery system on the viability of drug-resistant leukemia cell lines

The effects of different treatments on K562 R and K562 cells were analysed via Annexin V‒PI flow cytometry (apoptosis, Figures 4A,B) and an EdU proliferation assay (Figures 4C,D). In K562 R cells, the apoptotic rate in the IP@GLPs@εF group was significantly greater than that in the control groups (NC, LP, and GLP) and the ponatinib group. Moreover, it strongly inhibited K562 R proliferation, as indicated by a reduction in the number of EdU-positive cells. These results confirm that the dual-drug delivery system IP@GLPs@εF has a stronger inhibitory effect on drug-resistant leukemia K562R cells, which exhibit specific targeted cytotoxicity against drug-resistant strains.

### 3.5 Verification of the antiresistance efficacy of the dual-drug delivery system in an ectopic xenograft tumor model

**FIGURE 5:**
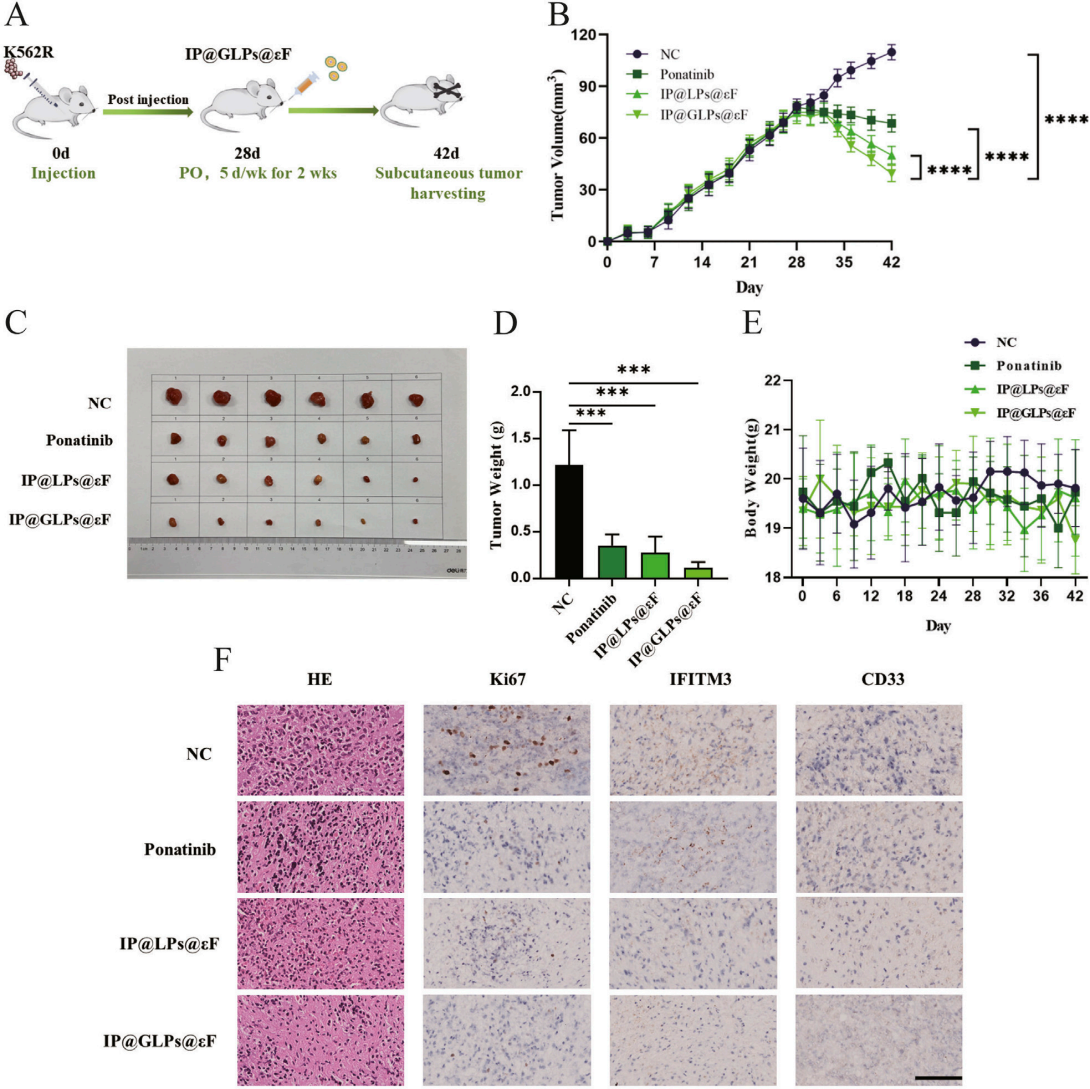
Antiresistance efficacy of the dual-drug system in K562R xenograft tumors. **(A)** Schematic illustration of the experimental procedure: K562R cells were subcutaneously inoculated into mice (Day 0), administration was initiated on Day 28 (oral gavage, 5 times per week for 2 consecutive weeks), and subcutaneous tumors were harvested on Day 42. **(B)** Tumor volume growth curves of each group over time (NC: negative control group; Ponatinib: single-agent control group). **(C)** Gross images of harvested tumors in each group. **(D)** Statistical analysis of the tumor weight in each group. **(E)** Body weight curves of the mice in each group over time. **(F)** Haematoxylin‒eosin (HE) staining and immunohistochemical (IHC) staining of tumor tissues for Ki67, IFITM3 and CD33. All the data represent the means ± SDs from three independent experiments. *p < 0.05, **p < 0.01, ***p < 0.001, ****p < 0.0001, N.S. P > 0.05.

The *in vivo* experimental procedure employed for mice bearing K562 R drug-resistant leukemia xenografts is presented in Figure 5A. Dynamic tumor volume monitoring (Figure 5B) revealed continuous rapid growth in the control group, whereas free drugs and vector controls resulted in only mild suppression. In contrast, the IP@GLPs@εF dual-drug-loaded system significantly inhibited K562R tumor growth, with a late-stage volume reduction and stronger *in vivo* antitumor activity. The tumor weight in the IP@GLPs@εF group was markedly lower than that in the other groups, further confirming the *in vivo* inhibitory effect of IP@GLPs@εF on K562 R cells (Figures 5C,D). Dynamic mouse body weight monitoring (Figures 5E) revealed no significant reduction in any group, indicating the low in vivo toxicity of the interventions. Haematoxylin–eosin (HE) staining and immunohistochemical staining (Figures 5F) revealed notably weaker Ki67, IFITM3, and CD33 signals in the IP@GLPs@εF group, suggesting that this system inhibits K562R cell proliferation and downregulates the expression of related proteins. Collectively, these results confirm that the IP@GLPs@εF dual-drug system has a more potent *in vivo* tumor-suppressive effect on K562R cells, with favourable biocompatibility and modulation of proliferation-associated proteins.

### 3.6 Therapeutic efficacy of the dual-drug delivery system against orthotopically implanted drug-resistant CML xenografts

**FIGURE 6:**
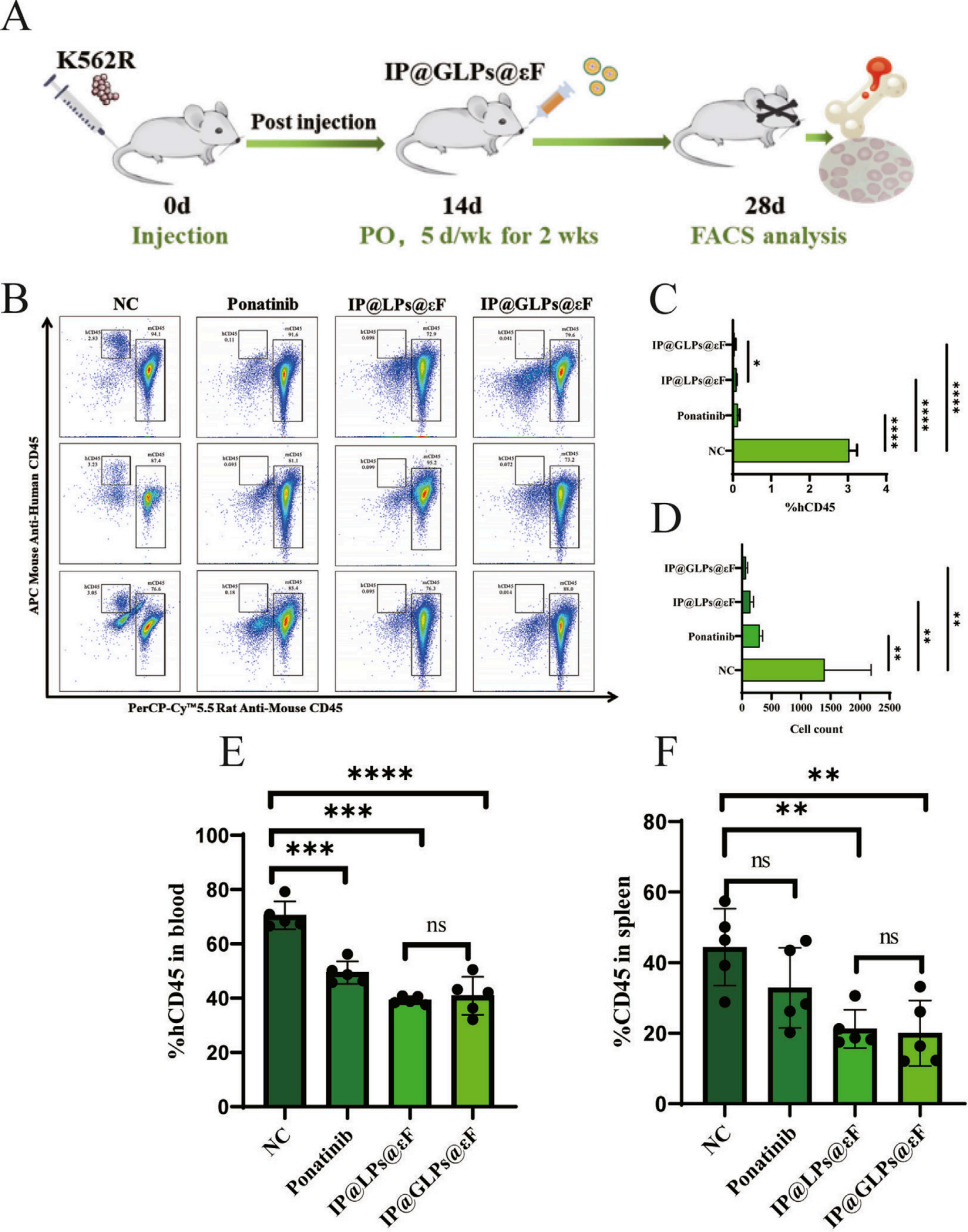
Therapeutic efficacy of the dual-drug system for drug-resistant CML xenografts. **(A)** Schematic illustration of the experimental procedure. **(B)** Representative flow cytometry plots. **(C,D)** Quantification of the proportion and absolute number of human CD45^+^ cells. **(E,F)** Quantification of the CML cell proportion in the peripheral blood and spleen. All the data represent the means ± SDs from three independent experiments. *p < 0.05, **p < 0.01, ***p < 0.001, ****p < 0.0001, N.S. P > 0.05.

To investigate the therapeutic effect of the dual-drug delivery system on orthotopically transplanted drug-resistant CML xenografts, K562 R cells were first inoculated into mice on Day 0. On Day 14, the mice were orally administered different formulations (5 times/week for 2 consecutive weeks) and divided into four groups: negative control (NC), ponatinib, IP@LPs@εF, and IP@GLPs@εF. Flow cytometry was performed on Day 28 (Figure 6A). Flow cytometry dot plots (Figure 6B) revealed a gradient decrease in the proportion of human CD45^+^ (hCD45^+^) cells in the ponatinib, IP@LPs@εF, and IP@GLPs@εF groups compared with that in the NC group (labeled with APC anti-human CD45^+^ antibody and PerCP-Cy™5.5 rat anti-mouse CD45^+^ antibody). Quantitative analysis (Figures 6C,D) confirmed that both CD45^+^ and hCD45^+^ levels were significantly decreased in the IP@GLPs@εF group. Consistent with the flow cytometry results, the IP@GLPs@εF group showed the most significant reduction in the hCD45^+^ cell proportion in mouse peripheral blood (Figure 6E) and the spleen (Figure 6F), indicating that this dual-drug delivery system may effectively inhibit the proliferation and dissemination of drug-resistant CML cells *in vivo*.

### 3.7 Silencing IFITM3 enhances the chemosensitivity of CML cells to tyrosine kinase inhibitors through direct inhibition of HSPA9

**FIGURE 7:**
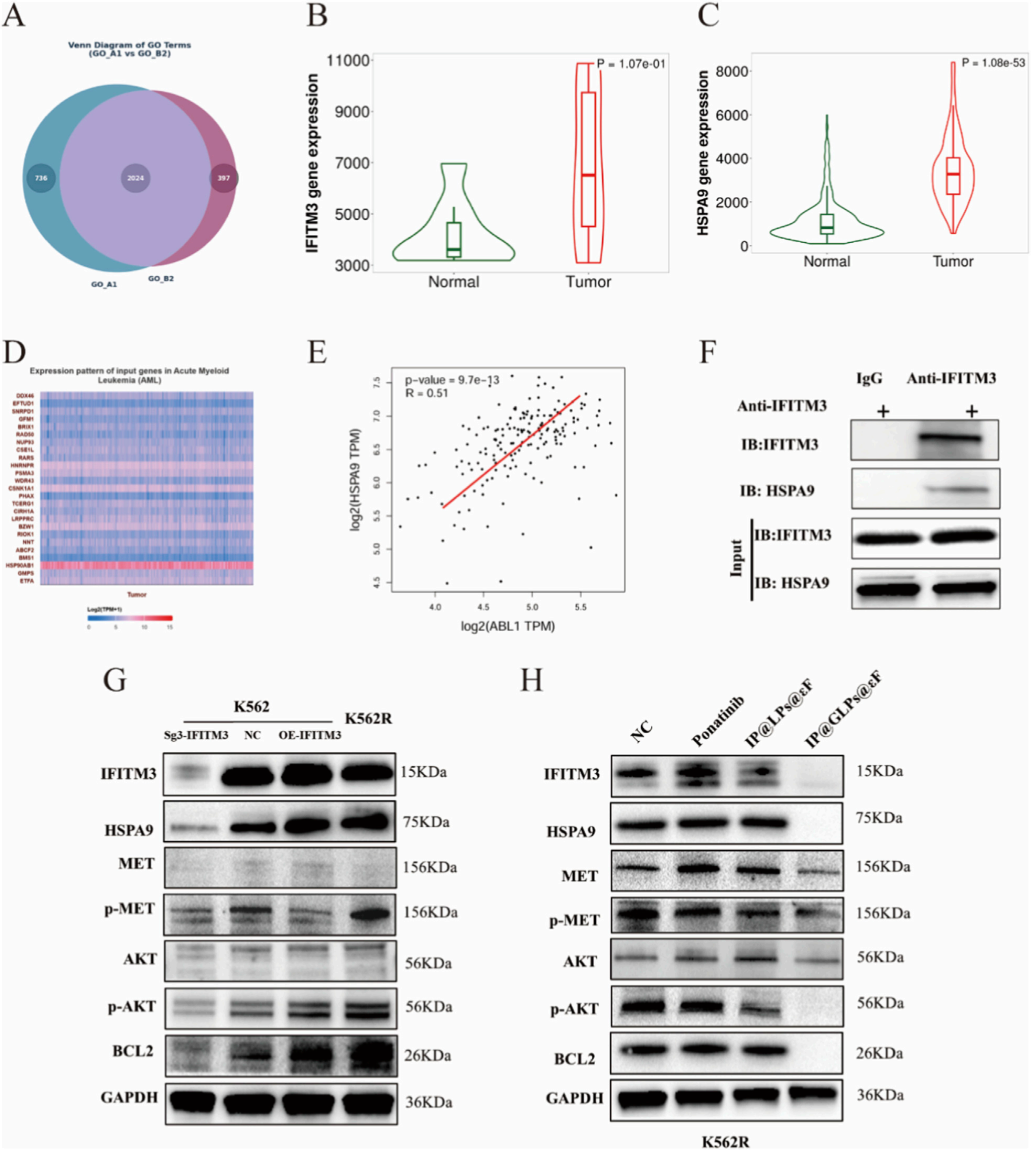
The IFITM3-HSPA9 axis regulates CML cell chemosensitivity to TKIs. **(A)** Venn diagram of GO terms for the GO_A1 (K562 R) and GO_B2 (K562) groups. **(B,C)** Box plots showing IFITM3 and HSPA9 expression in normal and tumor tissues. **(D)** Heatmap of IFITM3 expression in leukemia samples. **(E)** Scatter plot showing the correlation between IFITM3 and HSPA9 expression. **(F)** Validation of the IFITM3-HSPA9 interaction by coimmunoprecipitation. **(G)** Western blot analysis of IFITM3-regulated proteins in K562 and K562 R cells. **(H)** Western blot analysis of related proteins in K562R cells following drug intervention. All the data represent the means ± SDs from three independent experiments. *p < 0.05, **p < 0.01, ***p < 0.001, ****p < 0.0001, N.S. P > 0.05.

To explore the IFITM3 protein interaction network in drug-resistant cells, coimmunoprecipitation (Co-IP) with an anti-IFITM3 antibody was performed in parental K562 and drug-resistant K562 R cells. Mass spectrometry (MS) analysis of immunoprecipitated proteins revealed high enrichment of HSPA9 among IFITM3-interacting proteins in K562R cells (Figure 7A). Violin plots and heatmaps revealed that compared with normal samples, both IFITM3 and HSPA9 were highly expressed in leukemia samples (Figures 7B–D). Bioinformatics correlation analysis (scatter plot) revealed a moderate positive correlation between HSPA9 expression and ABL1 expression (R = 0.51) (Figure 7E). Co-IP in K562R cells confirmed a specific IFITM3–HSPA9 protein‒protein interaction (Figure 7F). Western blot analysis revealed that IFITM3 expression was downregulated in K562-sg3-IFITM3-knockdown cells, upregulated in K562-OE-IFITM3-overexpressing cells, and highly expressed in K562 R cells (comparable to overexpression levels). The expression of HSPA9 was consistent with that of IFITM3, verifying the interaction of these genes. Additionally, p-MET was markedly upregulated in K562 R cells, and p-AKT and antiapoptotic BCL2 levels increased synchronously with IFITM3 overexpression (Figure 7G). In xenograft tumor models, 2 weeks after treatment, Western blotting (Figure 7H) demonstrated that IP@GLPs@εF significantly reversed IFITM3-induced activation of the MET/AKT phosphorylation pathway and suppressed BCL2 expression. Concomitantly, IFITM3 and HSPA9 levels were reduced, indicating that the dual-drug treatment inhibited tumor cell anti-apoptosis by targeting the IFITM3-associated signaling axis.

### 3.8 Safety evaluation of the dual-drug delivery system

**FIGURE 8:**
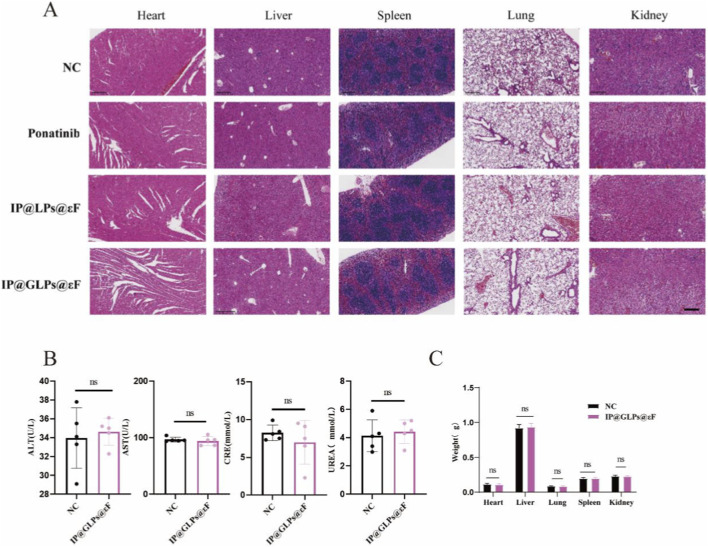
Safety evaluation of the dual-drug delivery system. **(A)** HE-stained sections of heart, liver, spleen, lung, and kidney tissues from mice in each group. **(B)** Results of the detection of serum liver and kidney function indicators (ALT, AST, CRE, and UREA). **(C)** Results of organ weight analysis. All the data represent the means ± SDs from three independent experiments (ns, P > 0.05, indicates no significant difference between groups).

*In vivo* safety assessment verified that this dual-drug delivery system possesses favorable biocompatibility and causes no obvious damage to the major organs of mice. HE staining of heart, liver, spleen, lung and kidney tissues from IP@GLPs@eF-treated mice revealed no obvious pathological damage, and the organ morphology and weight did not significantly differ between the IP@GLPs@eF-treated group and the NC group (Figures 8A,C). Moreover, serum indicators related to hepatic and renal functions (ALT, AST, CRE, and UREA) did not significantly differ from those in the NC group (ns) (Figure 8B), laying a critical safety foundation for the subsequent application of this delivery system.

## 4 Discussion

The core limitation in CML treatment is TKI resistance, and the high incidence of acquired resistance severely compromises long-term patient prognosis. In this study, we constructed a targeted nanodelivery system co-loading IFITM3 CRISPR/Cas9 knockout (IFITM3 KO) plasmid and ponatinib, achieving innovative synergism between gene editing and targeted therapy. Rational target selection represents a prerequisite for effective intervention. As a promising target for reversing TKI resistance in CML, IFITM3 is supported by both previous reports (Li et al., 2024; Cui Y. et al., 2025; Ibusuki et al., 2025; Chu et al., 2022; Xiong et al., 2024) and our current findings. Our preliminary work confirmed elevated IFITM3 expression in imatinib-resistant CML cells, and the present study further demonstrated that high IFITM3 expression correlates with reduced sensitivity to multiple TKIs. Mechanistically, we revealed that IFITM3 promotes MET phosphorylation and activation through HSPA9, thereby stimulating the AKT signaling axis and upregulating the anti-apoptotic protein BCL2. This cascade ultimately drives and sustains the TKI-resistant phenotype in CML cells.

Accordingly, we designed a combinatorial strategy based on “IFITM3 KO + ponatinib” to overcome the compensatory resistance frequently observed with single-agent targeted therapy, a key challenge in current CML research (Yuan et al., 2025; Busch et al., 2023; Yang et al., 2025; Tezcanli et al., 2024). In our system, IFITM3 KO blocks resistance signaling at the source, while ponatinib directly inhibits BCR-ABL kinase activity (including the T315I mutation) (Cortes et al., 2024; J et al., 2024). These two agents act through complementary mechanisms, yielding strong synergistic effects and markedly improving the killing efficiency against drug-resistant CML cells.

Combination regimens that integrate targeted drugs with gene-based interventions represent an effective strategy to enhance chemosensitivity in CML. RNA interference (RNAi) using siRNA has been widely explored in cancer gene therapy (Shi et al., 2017; Haussecker, 2014). However, RNAi suffers from inherent limitations, including inconsistent post-transcriptional silencing efficiency and off-target effects (Shi et al., 2017). In contrast, the CRISPR/Cas9 system enables precise, stable, and heritable gene disruption, holding greater potential for durable therapeutic efficacy in malignant diseases (Cui Z. et al., 2025; Kim et al., 2025; Masarwy et al., 2025; Zhang et al., 2024). Growing evidence indicates that CRISPR/Cas9-mediated gene editing combined with chemotherapy or targeted therapy achieves superior anti-tumor effects compared with monotherapy (Zhang et al., 2024; Koodamvetty and Thangavel, 2025).

Compared with conventional synthetic lipid nanoparticles and other natural biomaterial-based carriers, our ginger-derived lipid vector exhibits multiple distinctive advantages. Although chitosan, alginate, and plant-derived liposomes have been explored for drug delivery, most suffer from low structural stability, insufficient encapsulation efficiency for hydrophobic drugs and large plasmids, or limited biocompatibility. In contrast, ginger-derived lipids possess intrinsic biosafety, biodegradability, and low immunogenicity, while supporting high loading capacity for both small-molecule tyrosine kinase inhibitors and CRISPR/Cas9 plasmids. Unlike many natural carriers that require additional chemical modification to improve colloidal stability, our self-assembled ginger lipid system maintains uniform nanoparticle morphology and size distribution in aqueous environments. Furthermore, the integration of fucoidan modification enables specific targeting to P-selectin overexpressed on CML cells, a feature rarely achieved in unmodified natural lipid platforms. Collectively, this carrier combines the safety of edible natural materials with the performance of advanced delivery systems, addressing key limitations of existing natural-product-based nanocarriers (Srinivasan and Prasad, 2025; Chai et al., 2026; Sun L. et al., 2025; Xie et al., 2025).

We therefore engineered fucoidan-decorated ginger-derived lipid nanoparticles, which exhibited excellent stability, uniform spherical morphology, high encapsulation efficiency, and favorable drug-loading capacity. Fucoidan modification enabled specific binding to P-selectin on CML cells, significantly enhancing tumor-targeting efficiency. Future studies will further optimize targeting moieties (e.g., tumor-specific antibodies or aptamers) to improve lesion accumulation. We also plan to incorporate immune checkpoint inhibitors and additional resistance-related gene inhibitors to develop a multi-payload targeted nanosystem, thereby improving outcomes for patients with complex or multi-drug resistance.

Several limitations exist in this study. First, we only confirmed IFITM3 silencing rather than definitive gene knockout. Second, the K562 R cell model lacks detailed analysis of BCR-ABL1 mutations and bypass resistance mechanisms. Third, formal synergy analysis and complete control groups were insufficient. Fourth, the mechanism of the IFITM3-HSPA9-MET/AKT/BCL2 axis lacks rigorous rescue experiments. In addition, the oral delivery efficiency, *in vivo* targeting evidence, and long-term safety require further verification. These aspects will be improved in future research to enhance the reliability and translational value of our work.

Notably, TKI resistance in CML is highly heterogeneous and involves diverse genetic alterations as well as tumor microenvironment-mediated non-cell-autonomous mechanisms. Accordingly, a single dual-loading system cannot cover all resistance subtypes, representing an important direction for further optimization.

## Data Availability

The original contributions presented in the study are included in the article/supplementary material, further inquiries can be directed to the corresponding authors.
